# Crystal Structure, Antibacterial and Cytotoxic Activities of a New Complex of Bismuth(III) with Sulfapyridine

**DOI:** 10.3390/molecules18021464

**Published:** 2013-01-24

**Authors:** Ivana M. Marzano, Marina S. Franco, Priscila P. Silva, Rodinei Augusti, Geandson C. Santos, Nelson G. Fernandes, Mônica Bucciarelli-Rodriguez, Edmar Chartone-Souza, Elene C. Pereira-Maia

**Affiliations:** 1 Department of Chemistry, Universidade Federal de Minas Gerais, Av. Antônio Carlos, 6627, Belo Horizonte 31270-901, MG, Brazil; 2 Department of General Biology, Universidade Federal de Minas Gerais, Av. Antônio Carlos, 6627, Belo Horizonte 31270-901, MG, Brazil

**Keywords:** Bi(III) complex, sulfapyridine, X-ray structure, antimicrobial activity, cytotoxic activity

## Abstract

A new complex of Bi(III) and sulfapyridine was synthesized and characterized by elemental analysis, atomic absorption spectrometry, conductivity analysis, electrospray ionization mass spectrometry (ESI-MS), infrared spectroscopy, and single crystal X-ray diffraction methods. The antimicrobial and the cytotoxic activities of the compound were investigated. Elemental and conductivity analyses are in accordance to the formulation [BiCl_3_(C_11_H_11_N_3_O_2_S)_3_]. The structure of the complex reveals a distorted octahedral geometry around the bismuth atom, which is bound to three sulfonamidic nitrogens from sulfapyridine, acting as a monodentate ligand, and to three chloride ions. The presence of the compound in solution was confirmed by ESI-MS studies. The complex is 3 times more potent than the ligand against *Salmonella typhimurium*, 4 times against *Staphylococcus aureus*, *Shigella dysenteriae*, and *Shigella sonnei* and 8 times more potent against *Pseudomonas aeruginosa* and *Escherichia coli*. The compound inhibits the growth of chronic myelogenous leukemia cells with an IC_50_ value of 44 μM whereas the free ligand has no effect up to 100 μM.

## 1. Introduction

The excessive use of antibiotics caused the emergence of drug-resistant bacterial strains through a variety of mechanisms. This problem has become quite alarming because almost all pathogenic bacteria have acquired resistance against the presently known and widely used antibiotics [1,2,3].

Sulfonamides were the first synthetic antibiotics to be used in clinic and they exhibit interesting pharmacological properties, such as selectivity to bacterial cells and low toxicity. Sulfapyridine was the agent of choice for the treatment of pneumonia for a time. The mode of action of sulfonamides involves the disruption of bacterial folic acid synthesis, which is essential for the biosynthesis of *N*-methylated pyrimidines and purines as well as certain aminoacids, that are, in turn, necessary for bacterial growth. Sulfapyridine mimics the paraaminobenzoic acid (PABA), which is a natural substrate required for the biosynthesis of folic acid. More specifically, sulfapyridine inhibits the activity of the enzyme dihydropteroate synthase (DHPS), which catalyzes the displacement of pyrophosphate from a pteridine substrate resulting in a covalent bond to the amino group of PABA. The competition between sulfapyridine and PABA for the enzyme results in sulfapyridine binding, because it possesses a greater affinity for the enzyme. This inhibits the enzyme activity and consequently the biosynthesis of folic acid [4]. Mammals obtain folic acid from food and are unable to synthesize it. Because of this, sulfonamides are selective agents to bacteria and well tolerated by humans. Unfortunately, resistance to sulfonamides, mainly due to the presence of drug-resistant variants of the DHPS enzymes, limits its clinical use [5].

Bismuth compounds have been used for centuries in the treatment of a variety of microbial infections due to their high efficacy and low toxicity. One can cite their use in the treatment of parasitic infections, gastric disorders, and peptic ulcers. More recently, the discovery that bismuth inhibits the growth of *Helicobacter pylori* and is effective in eradicating this bacterium when administered in combination with antibiotics rehabilitated bismuth compounds in chemotherapy [6,7]. The bismuth drugs most used in the treatment of gastrointestinal diseases related to the infection of Helicobacter pylori are bismuth subsalicylate, coloidal bismuth subcitrate and ranitidine bismuth citrate [8,9,10,11]. However, the pharmaceutical formulations are not very well defined or stable, bismuth compounds are not very soluble and the active species are not well characterized. The lability of the ligands, the Lewis acidity and ability to expand the coordination sphere of the metal center make the control of the reactions and characterization of the complexes a difficult task. A way of attenuating the hydrolysis of bismuth complexes in aqueous solution with the formation of the [BiO^+^] cation is the incorporation of N, O or S donor atoms into coordinating ligands. [12]. These aspects have stimulated the search for new bismuth drugs presenting antibacterial activity, such as compounds containing non-steroidal anti-inflammatory drugs [13], ciprofloxacin [14,15], thiosemicarbazones [16,17,18,19,20,21] or lipophilic thiols [22]. It is worth notifying that the bismuth complex with 2-acetylpyrazine *N*(4)-pyridylthiosemicarbazone described by Li-Zhi Zhang *et al.* exhibited effective antibacterial and anticancer activities [21]. More recently, P.C. Andrews *et al.* prepared some tris-substituted bismuth(III) sulfonates, which exhibited MIC (minimal inhibitory concentration) values in the nanomolar range against *Helicobacter pylori* [23].

The mechanism of the antibacterial action of bismuth drugs is not yet completely understood and seems to involve enzyme inhibition. Bismuth inhibits fumarase (that catalyses the hydration of fumarate to malic acid in the bacterial tricarboxylic acid cycle), cytosolic alcohol dehydrogenase (ADH production of acetaldehyde), inhibits urease (that converts urea into ammonia and carbonic acid), which is crucial for *H. pylori* survival in the low pH of stomach, induces oxidative stress and a decrease in cellular protease activities [24,25].

The emergence of multidrug resistance to almost all known antibiotics makes the discovery of new agents a crucial necessity. We have been working on the coordination of antibiotics to metal ions as a promising strategy to obtain more active agents [26,27,28]. Our strategy is to ally the pharmacological properties of bismuth compounds to those of sulfonamides in the search for better chemotherapeutic compounds. A favorable characteristic of bismuth drugs is that there is no evidence of resistance as observed with standard antibiotic compounds. We have synthesized, characterized and studied the antimicrobial and cytotoxic activities of a new compound of bismuth(III) and sulfapyridine.

## 2. Results and Discussion

A new complex of Bi(III) with sulfapyridine was synthesized and characterized by elemental analysis, atomic absorption spectrometry, conductivity analysis, electrospray ionization mass spectrometry (ESI-MS), infrared spectroscopy, and single crystal X-ray diffraction methods.

**Figure 1:**
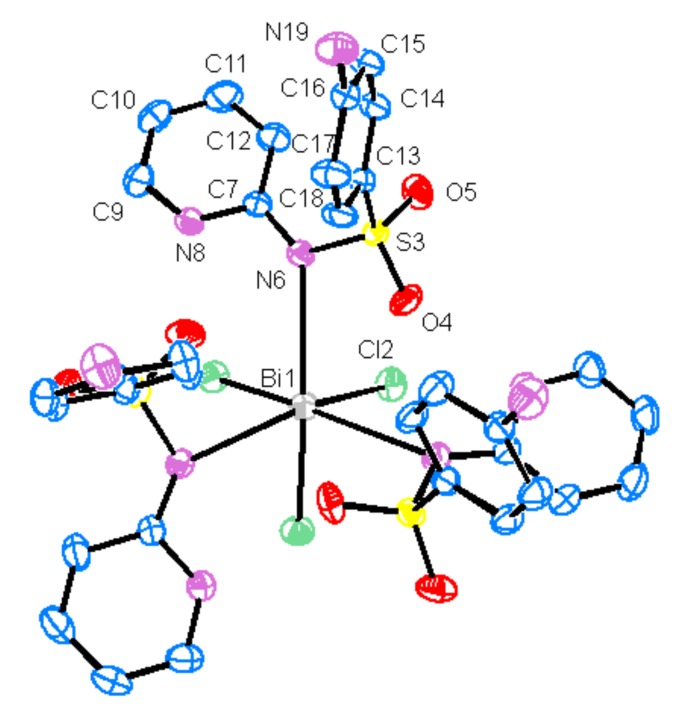
The molecular structure and the atom numbering scheme. Ellipsoids are drawn to include 50% probability, the hydrogen atoms have been omitted.

Single-crystal X-ray analysis reveals that the complex crystallizes in the trigonal system, with space group R 3. Crystallographic data and details of crystal data, data collection and refinement are summarized in Table 1. Selected bond lengths, angles and geometrical parameters for hydrogen bonds are reported in Table 2. As can be seen in Figure 1, the molecular structure of [BiCl_3_(C_11_H_11_N_3_O_2_S)_3_] shows bismuth bound to three sulfapyridine molecules and three chloride ions in a distorted octahedral environment. By symmetry imposition the three Cl2-Bi1-Cl2 angles are equal to each other, 87.74(3)°, as well as the N6-Bi1-N6 angles, 108.79(5)°, however, they differ from the idealized octahedral geometry, mainly the N6-Bi1-N6 angles. This distortion can be caused by steric and electronic effects of the ligands combined with the presence of the bismuth(III) lone pair of electrons (6s^2^). The neutral sulfapyridine ligands (deprotonated in the sulfonamidic nitrogen and protonated in the pyridinic nitrogen) are coordinated to bismuth(III) in a unidentate way *via* its sulfonamidic nitrogen (N6). The H8 atom has been found in the difference map, however, no significant differences can be found in the C7-N6, C7-N8 and C9-N8 distances, which indicates that C7-N6 and C7-N8 bonds have the same bond order. The 3+ charge of bismuth is neutralized by the chloride ions, being the Bi1-Cl2 bond lengths 2.5442 Å, which falls within the range acceptable for other complexes [14,15,29]. The Bi1-N6 distance is 2.924 Å, which reveals an interaction of medium strength, the sum of covalent and van der Waals radii for nitrogen and bismuth are Σrcov(Bi,N) 2.27 Å and ΣrvdW(Bi,N) 3.94 Å, respectively [29]. A Bi-N distance of 2.9(1) Å was also found in a similar compound, the trichlorotris-(3-sulphanilamido-6-methoxypyridazine)-bismuth(III) [30]. Longer Bi-N distances were found for some distorted octahedral organobismuth(III) compounds possessing a (C,N)_3_Bi core: in [2-{O(CH_2_CH_2_)_2_NCH_2_}C_6_H_4_]_3_Bi, B-N = 3.170(7) Å, in [2-{MeN(CH_2_CH_2_)_2_NCH_2_}C_6_H_4_]_3_Bi, B-N = 3.211(5) Å [27] and in [2-(Et_2_NCH_2_)C_6_H_4_]_3_Bi, Bi-N = 3.214(7) Å. [31].

**Figure 2:**
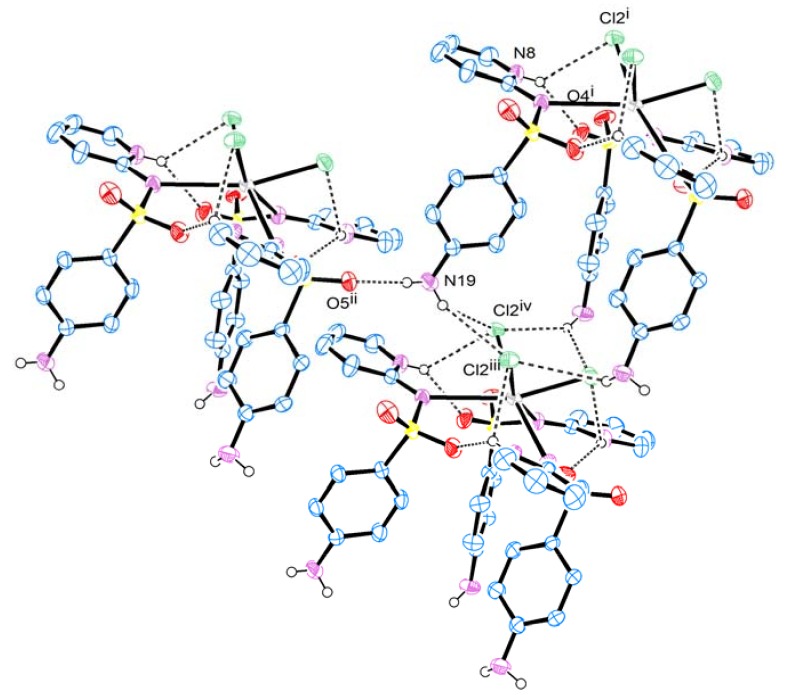
The tridimensional network of hydrogen bonds. Ellipsoids are drawn to include 50% probability, the hydrogen atoms which are not involved in hydrogen bonds have been omitted.

Figure 2 shows the tridimensional network of hydrogen bonds which contribute to the crystal structure stabilization of the title complex. There are three intermolecular hydrogen bonds, two of them are bifurcated hydrogen bonds: N8-H8···O4(Cl2) and N19-H19B···Cl2(Cl2). On the other hand, N19-H19A···O5 is almost linear, as shown in Figure 2 and Table 2.

The molar conductivity value of 10^−3^ M solution in dimethylformamide at 25 °C was 10.35 Ohm^−1^ cm^2^ mol^−1^, which indicates that the complex is neutral. The literature values for a 1:1 electrolyte in dimethylformamide are in the range 65–90 Ohm^−1^ cm^2^ mol^−1^ [32].

The modifications observed in the infrared spectrum of the complex in comparison to that of the free ligand are in accordance with the coordination via the sulfonamidic nitrogen (Figure S1). The spectrum of the complex is significantly modified in the region 3400–3000 due to the deprotonation of the sulfonamidic nitrogen and protonation of the pyridinic nitrogen. In the sulfapyridine spectrum two bands at 3418 and 3312 cm^−1^ are assigned to νas(NH_2_) and νs(NH_2_), respectively. Coordination to Bi(III) shifts these absorptions to 3423 and 3333 cm^−1^. It is difficult to verify the deprotonation of the sulfonamidic nitrogen because of the concomitant protonation of the pyridinic nitrogen in the complex, which leads to the appearance of multiple absorptions in the region 3300–2960 cm^−1^ [33]. The asymmetric and symmetric –SO_2_ group stretching vibrations in the sulfapyridine spectrum appear at 1369 and 1126 cm^−1^, respectively. The νas (SO_2_) shifted from 1369 cm^−1^ in the free ligand to 1346 cm^−1^ in the complex, due to the deprotonation of the sulfonamidic nitrogen and bismuth coordination, while the νs(SO_2_) remained almost unchanged. The same effect was observed in the infrared spectra of other sulfonamide complexes, in which coordination sites did not involve the SO_2_ group [34,35,36].

The presence of the complex in solution was confirmed by ESI-MS studies. In the mass spectrum, positive mode, a peak at *m/z* 1063 refers to the molecular ion of the complex, *i.e.*, [BiCl3(C11H11N3O2S)3]^+●^ (M^+●^), which arises from the oxidation (removal of one electron) of the neutral species [Figure S2]. The mass-selection and fragmentation (by collision induced dissociation) of this ion yields an intense peak at *m/z* 815, whose structure is suggested to be formally [BiCl_3_(C_11_H_11_N_3_O_2_S)_2_ + H]^+^. This product ion is proposed to be generated via a net loss of [C_11_H_10_N_3_O_2_S]^●^ (sulfapyridine-H^●^) from the molecular ion.

The main modification observed in the ^1^H-NMR spectrum of the complex in comparison to that of the free ligand (Figure S3) is the absence of a resonance at δ 10.8 assigned to the proton of the sulfonamidic nitrogen, indicating its deprotonation.

We have first checked that the solvent does not present an antibacterial effect in the concentrations used. The antibacterial effect of the complex and the ligand was studied by determining the minimal inhibitory concentration, MIC, which is the minimal concentration required to completely inhibit bacterial growth. The complex inhibited the bacterial growth in a concentration-dependent way. The MIC values obtained in six bacterial strains resistant to sulfapyridine are shown in Table 3. The MIC of sulfapyridine against *Escherichia coli* was not attained up to the highest concentration tested (1,027 μM), while for the complex the MIC value was 241 μM. The most important result is that the complex is 3 times more potent than the ligand against *Salmonella typhimurium*, 4 times against *Staphylococcus aureus*, *Shigella dysenteriae*, and *Shigella sonnei* and 8 times against *Pseudomonas aeruginosa*.

There are few works reporting on the study of metal compounds with sulfonamides. Kremer *et al.* [34] studied the antibacterial activities of compounds with sulfonamides and Cu(II) ions. The most important result was obtained with the compound [Cu(sulfametoxazol)_2_(H_2_O)_4_]∙2H_2_O, which was 4 times more active than the ligand against *S. aureus* and *E. coli.* The authors suggested that the improvement in the antimicrobial activity could be related to the higher lipophilicity of the complexes in relation to free sulfonamides, which would increase their uptake in bacterial cells.

Mondelli *et al.* [35] synthesized and characterized a Ni(II) compound of sulfapyridine, in which nickel is in a distorted octahedral environment, coordinated by two aryl amine N from two sulfonamides acting as monodentate ligands and four N atoms (two sulfonamidic N and two heterocyclic N) from two different sulfonamide molecules acting as bidentate ligands. Unfortunately, the complex was less active against *E. coli* and *S. aureus* than the free ligand. The authors proposed that a reduced uptake could be the reason for the lower activity.

The title complex is more active than the free ligand in six bacterial strains resistant to sulfapyridine. This is an important result because bacterial resistance is the major obstacle for the treatment of bacterial infections.

The fact that some bismuth compounds, such as tropolone or thiosemicarbazones complexes and bismuth(III) dithiocarbamates present antitumor activities [19,37,38] motivated us to study the effect of the synthesized complex in the growth of tumoral cells. The mechanism of the cytotoxic action of some bismuth-containing drugs has been suggested to involve inhibition of enzymes, such as proteases, lipases, glycosidases and phospholipases [39].

The sensitivity of chronic myelogenous leukemia to the compounds was evaluated by incubating cells for three days in the presence of increasing complex concentration. Afterwards, cells were counted and the concentration required to inhibit 50% of cell growth was calculated, the IC_50_.

**Figure 3:**
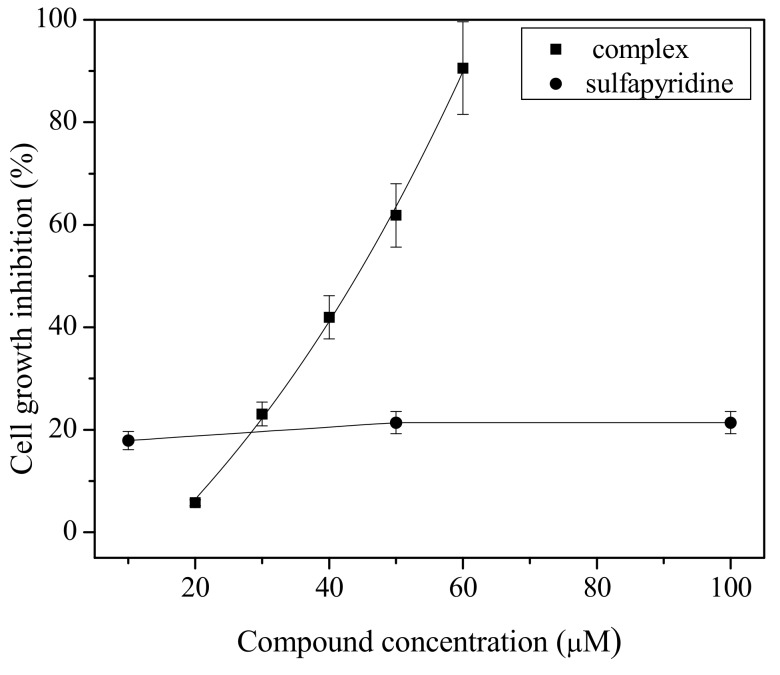
Cell growth inhibition by [BiCl_3_(C_11_H_11_N_3_O_2_S)_3_] and sulfapyridine. Cells were incubated with compounds for three days.

The compound inhibited the growth of K562 cells with the IC_50_ value of 44 μM while sulfapyridine was not active up to 100 μΜ (Figure 3). Mammalian cells do not have the enzyme dihydropteroate synthase, which is the pharmacological target of sulfapyridine. Therefore, the cytotoxic action of the compound should be related to bismuth(III) coordination.

## 3. Experimental

### 3.1. General and Instruments

Sulfapyridine (4-amino-*N*-pyridin-2-ylbenzenesulfonamide) and bismuth chloride were purchased from Sigma Co. (St. Louis, MO, USA) All other chemicals were reagent-grade and were used without further purification. Infrared spectra were recorded in KBr pellets over the region 400–4000 cm^−1^ with a Perkin-Elmer 283 B spectrometer (Perkin Elmer Inc., Waltham, MA, USA). Full scan mass spectra were obtained on a LCQ Fleet mass spectrometer from ThermoScientific (San Jose, CA, USA) equipped with an electrospray source operating in the positive ion mode. Samples were dissolved in MeOH containing 0.1% formic acid and were injected in the apparatus by direct infusion at a flow rate of 10 μL min^−1^. The major ESI source conditions were: scan range 100–2,000 *m/z*, capillary temperature 275 °C, spray voltage 25 kV, capillary voltage 25 V, and sheath gas (N_2_) flow rate 10 units. For ESI-MS^2^, the precursor ions of interest were first isolated by applying an appropriate waveform across the end cap electrodes of the ion trap to resonantly eject all trapped ions except those of *m/z* ratio of interest. The isolated ions were then resonantly excited with a supplementary AC signal to cause collision-induced dissociation (CID). The relative collision energy was set to a value at which the ions were produced in measurable abundance.

Single crystals of [BiCl_3_(C_11_H_11_N_3_O_2_S)_3_] suitable for an X-ray analysis were grown by slow evaporation from an ethanolic solution and were obtained as yellow trigonal prismatic crystals. Single-crystal X-ray diffraction data were collected at 293 K on an Oxford Gemini Atlas Ultra diffractometer with graphite-monochromated, λ (MoKα) = 0.71073 Å using the CrysAlis-Pro data collection and data processing software [40]. The structure was solved by the SIR97 program 1 [41] incorporated in the WinGX program package [42]. All non hydrogen atoms were refined anisotropically on F^2^ using full matrix least-square procedure [SHELXL-97] [43] with weight: *w* = 1/[σ2(*F*o2) + (0.0347*P*)2], where *P* = (*F*o2 + 2*F*c2)/3. The hydrogen atoms were found in the difference Fourier maps and were refined using a riding model. A total of 6528 reflections have been measured and 4005 independent reflections were obtained. *R*_int_ = 0.024, 172 parameters refined, *R*[*F*^2^ > 2σ(*F*^2^)] = 0.023, *wR*(*F*^2^) = 0.055, *S* = 1.00, (Δ/σ)_max_ = 0.001, Δρ_min_ = −0.55, Δρ_max_ = 1.37 e Å^−3^ at 0.74 Å from Bi1 site. The absolute structure was determined giving a Flack parameter of 0.03(3) [44]. The structures were drawn by Mercury [45] and ORTEP-3 for windows [46] programs.

X-ray crystallographic data: CCDC No. 828007 contains the supplementary crystallographic data for this paper. These data can be obtained free of charge via www.ccdc.cam.ac.uk/conts/retrieving.html (or from the CCDC, 12 Union Road, Cambridge CB2 1EZ, UK; Fax: +44 1223 336033; E-Mail: deposit@ccdc.cam.ac.uk.

Carbon, nitrogen and hydrogen analysis were determined on a Perkin-Elmer 2400 CHN. Atomic absorption analysis of the bismuth content was carried out on a model 8200 Hitachi Atomic Absorption Spectrophotometer (Hitachi Ltd., Tokyo, Japan). Conductivity measurements were carried out with a Digimed DM 31 (Digimed, São Paulo, Brazil) conductivity meter using a cell of constant 1.023 cm^−1^. The solvent used was spectroscopic grade dimethylformamide (Merck, Darmstadt, Germany) (Λ_M_ = 4.08 Ohm^−^^1^ cm^2^ mol^−^^1^).

The melting point/decomposition temperature was determined on a MQAPF-302 (Microquímica Equipamentos Ltda, Palhoça, Brazil) instrument.

### 3.2. Synthesis

Sulfapyridine (*p*-amino-*N*-(2-pyridyl)benzenesulfonamide) (0,142 g or 5.7 × 10^−4^ mol) was dissolved in water at pH 9 (the pH was adjusted with NaOH) and bismuth chloride (0.060 g or 1,9 × 10^−4^ mol) was dissolved in water at pH 1 (the pH was lowered with HCl). The sulfapyridine solution was added dropwise to that of BiCl_3_, under continuous stirring. A pale yellowish solid was formed and separated by filtration, washed with water and dried under vacuum. The complex is soluble in dimethylformamide, dimethyl sulfoxide and slightly soluble in ethanol or methanol. Yield: 0.107 g, 53%. Anal. calc. for [Bi Cl_3_(C_11_H_11_N_3_O_2_S)_3_]: %C, 37.28; %H, 3.14; %N, 11.86; %Bi, 27.46. Found: C, 37.99; H, 3.18; N, 11.84; Bi, 27.98%. m.p. 505 K (decomposition). IR (cm^−1^): 3423s; 3333s; 3244m; 3211sh; 3148w; 3111w; 3087w; 3059w; 3026w; 2959w; 1628s; 1591s; 1529s; 1501s; 1464w; 1379s; 1346s; 1300s; 1279s; 1255s; 1241s; 1184w; 1167w; 1126s; 1082s; 1009m; 964s; 881w; 839m; 821w; 797s; 772m; 725w; 677s; 644w; 610w.

### 3.3. Microbial Strains and Growth Conditions

The bacterial strains selected from the bacteria collection of the Laboratory of Microbial Molecular Genetics of the Department of General Biology, ICB-UFMG, to perform microbiological tests were: *E. coli* ATCC 25922, *S. aureus* ATCC 25923, *P. aeruginosa* ATCC 24853, *S. typhimurium* ATCC 13311, *S. sonnei* ATCC 11060, *S. dysenteriae* ATCC 13313. Bacterial cultures were grown in the medium specified at 37 °C. Culture stocks were performed on Lignières medium (0.8% w/v Difco Nutrient Broth, 0.5% w/v Sigma gelatin, 0.7% w/v Difco agar).

### 3.4. Determination of Minimal Inhibitory Concentration

Sulfapyridine and the compound [BiCl_3_(C_11_H_11_N_3_O_2_S)_3_] were dissolved in *N,N*-dimethylformamide. Stock solutions were diluted accordingly and added to Mueller Hinton Agar (Difco) previously melted and cooled to 40 °C for the preparation of antibiotic plates. This agar was distributed onto Petri dishes so as to obtain sulfapyridine (sp) concentrations of 8, 16, 32, 64, 96, 128, 193, 257, 385, 513 and 1,027 μM, or [BiCl_3_(sp)_3_] concentrations of 2, 4, 8, 15, 23, 45, 60, 90, 120 and 241 μM. Plates for each concentration were prepared in quadruplicate. Control plates containing only the solvent (dimethylformamide) or Mueller Hinton Agar (Difco) without either drug or solvent were also prepared.

The bacterial strains selected were transferred to 2.0 mL of the Brain Heart Infusion (Difco) and incubated at 37 °C for 24 h. The resulting cultures were diluted 100 times and inoculated onto the plates previously prepared with antibiotics by means of a multi-inoculation apparatus (steer type) and were incubated at 37 °C for 24 h. Bacterial growth was recorded after this period.

### 3.5. Cell Line and Culture

The K562 cell line was purchased from the Rio de Janeiro Cell Bank (number CR083 of the RJCB collection). This cell line was established from pleural effusion of a 53 year-old female with chronic myelogenous leukemia in terminal blast crisis. Cells were cultured in RPMI 1640 (Sigma Chemical Co.) medium supplemented with 10% fetal calf serum (CULTILAB, São Paulo, Brazil) at 37 °C in a humidified 5% CO_2_ atmosphere. Cultures grow exponentially from 10^5^ cells mL^−1^ to about 8 × 10^5^ cells mL^−1^ in three days. Cell viability was checked by Trypan Blue exclusion. The cell number was determined by Coulter counter analysis.

### 3.6. Cytotoxicity Assays

For cytotoxicity assessment, 1 × 10^5^ cells mL^−1^ were cultured for 72 h in the absence and the presence of various concentrations of the tested compounds. The sensitivity to drug was evaluated by the concentration that inhibits cell growth by 50%, IC_50_.

## 4. Conclusions

A new complex of Bi(III) with sulfapyridine was synthesized and its crystal structure was determined. [BiCl_3_(C_11_H_11_N_3_O_2_S)_3_] was more active than the free ligand in six bacterial strains resistant to sulfapyridine. In addition, the compound inhibited the growth of chronic myelogenous leukemia cells while sulfapyridine was not active. Therefore, bismuth coordination enhances both antibacterial and cytotoxic action.

## Figures and Tables

Symmetry codes: (i) −y, x − y, z; (ii) –x + y + 1/3, −x + 2/3, z + 2/3; (iii) x, y, z + 1; (iv) −y, x−y, z + 1.

**Table 1:** Crystal data, data collection and structure refinement details for [BiCl_3_(C_11_H_11_N_3_O_2_S)_3_].

Empirical formula	C_33_H_33_BiCl_3_N_9_O_6_S_3_
Formula weight	1063.22
Temperature	293(2) K
Wavelength	0.71073 Å
Crystal system	Trigonal
Space group	R 3
Unit cell dimensions	a = 17.9557(3), b = 17.9557(3), c = 10.19691 (12) Å, α = 90, β = 90, γ = 120°
Volume	2847.12(7) Å^3^
Z	3
Density (calculated)	1.860 Mg/m^3^
Absorption coefficient	5.08 mm^−1^
F(000)	1572
Crystal size	0.23 × 0.25 × 0.29 mm^3^
Theta range for data collection	4.00 to 37.9°
Index ranges	−27 ≤ h ≤ 30, −29 ≤ k ≤ 15,−9 ≤ l ≤ 17
Reflections collected	6528
Independent reflections	4005(R_int_ = 0.024)
Absorption correction	Empirical
Refinement method	Full-matrix least-squares on F^2^
Data/restraints/parameters	4005/4/172
Goodness-of-fitonF^2^	1.00
Final R indices (for 3857 reflections with *I* > 2σ(*I*))	R1 = 0.023, wR2 = 0.055
R indices (all data)	R1 = 0.024, wR2 = 0.057
Largest diff. peak and hole	1.37 and −0.55 eÅ^−3^
Absolute structure [5]	Flack parameter = 0.003(3)

**Table 2:** Selected bond lengths and hydrogen bonding geometries (Å, °).

Bi1—Cl2	2.5442 (7)	Bi1—N6		2.924 (2)	
C7—N6	1.349 (4)	C7—N8		1.359 (4)	
C9—N8	1.344 (4)	C16—N19		1.383 (4)	
*D*—H···*A*	*D*—H	H···*A*	*D*···*A*		*D*—H···*A*
N8—H8···O4 ^i^	0.86	2.13	2.858 (3)		142
N8—H8···Cl2 ^i^	0.86	2.81	3.340 (3)		122
N19—H19A···O5 ^ii^	0.85 (1)	2.14 (1)	2.995 (4)		177 (5)
N19—H19B···Cl2 ^iii^	0.86 (1)	2.83 (4)	3.568 (3)		146 (5)
N19—H19B···Cl2 ^iv^	0.85(1)	3.12 (4)	3.702 (3)		127 (4)

Symmetry codes: (i) −y, x − y, z; (ii) –x + y + 1/3, −x + 2/3, z + 2/3; (iii) x, y, z + 1; (iv) −y, x − y, z + 1.

**Table 3:** MIC of free sulfapyridine (sp) and the bismuth (III) complex.

Bacterial strains		MIC ^a^/µM	
		sp	[Bi(sp)_3_Cl_3_]
*E. coli*	ATCC 25922	>1027	241
*S. aureus*	ATCC 25923	385	90
*P. aeruginosa*	ATCC 24853	1027	120
*S. typhimur ium*	ATCC 13311	96	30
*S. sonnei*	ATCC 11060	1027	241
*S. dysenteriae*	ATCC 13313	128	30

^a^ MIC is the minimal drug concentration required to inhibit bacterial growth. Number of assays = 4.

## Supplementary Materials

Supplementary materials can be accessed at: http://www.mdpi.com/1420-3049/18/2/1464/s1.
