# Supplementary material for: Crystal Structure, Antibacterial and Cytotoxic Activities of a New Complex of Bismuth(III) with Sulfapyridine
Source: Molecules. 2013 Jan 24;18(2):1464–76. doi: 10.3390/molecules18021464 (PMC6270105; doi:10.3390/molecules18021464)

**Supporting Information:** Figures S1, S2, and S3.

**Figure S1.** The infrared spectra of sulfapyridine (top) and its Bi(III) complex (bottom), [Bi(sp)$_3$Cl$_3$].

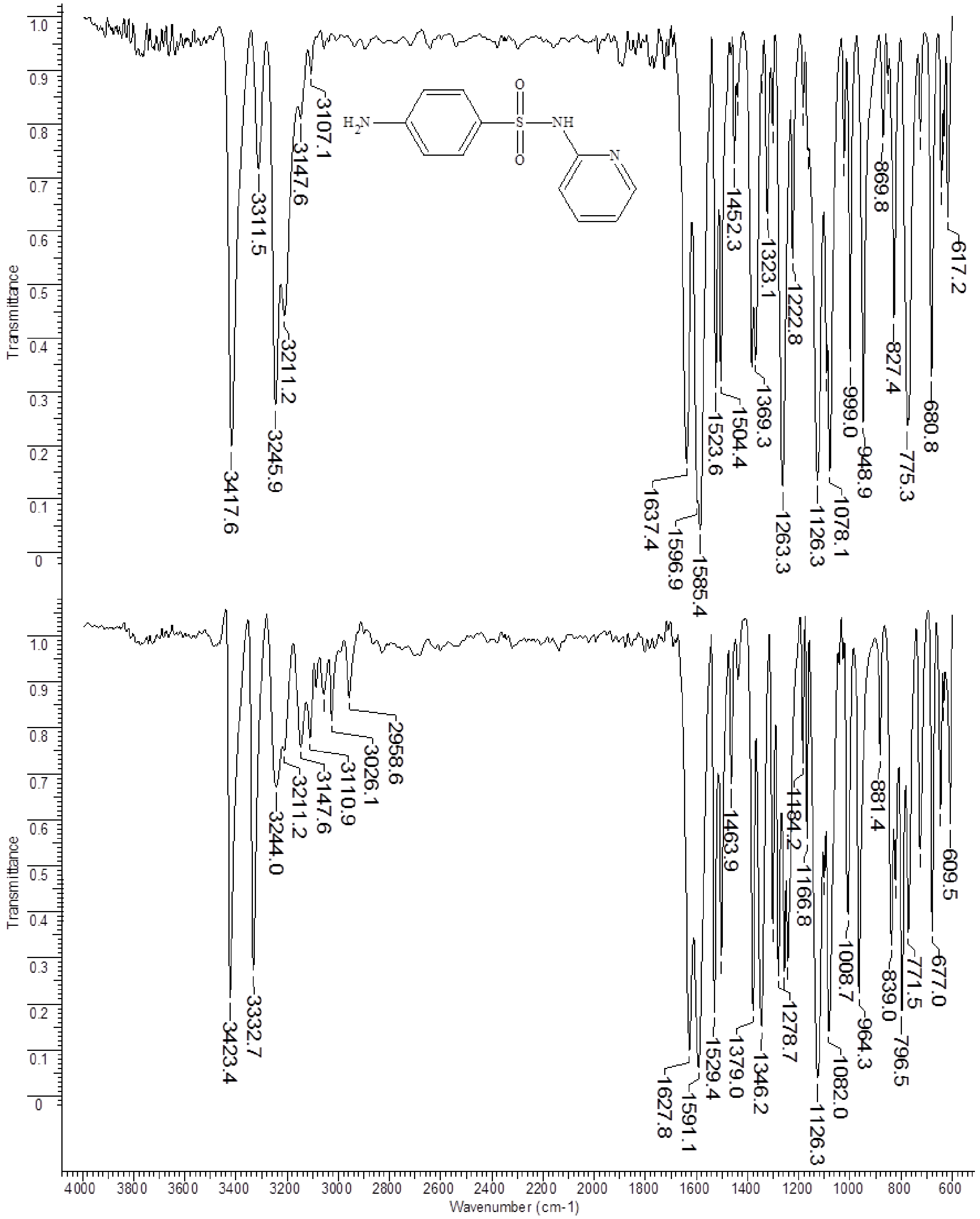

**Figure S2.** Expanded view of the ESI(+)-MS of the molecular ion of the complex, $[BiCl_3(C_{11}H_{11}N_3O_2S)_3]^{+\bullet}$ ($M^{+\bullet}$). The complex was dissolved in MeOH containing 0.1% formic acid.

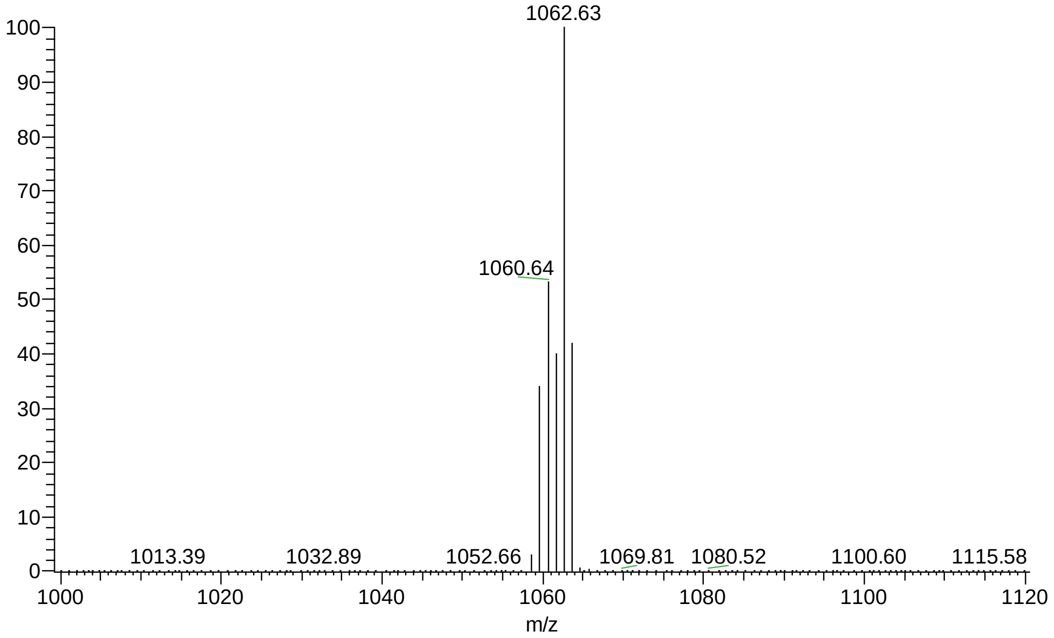


**Figure S3.** $^{1}$H-NMR spectra of sulfapyridine (top) and its Bi(III) complex $[Bi(sp)_3Cl_3]$ (bottom). The solvent is DMF-$d_7$ (*N*,*N*-dimethylformamide-$d_7$).

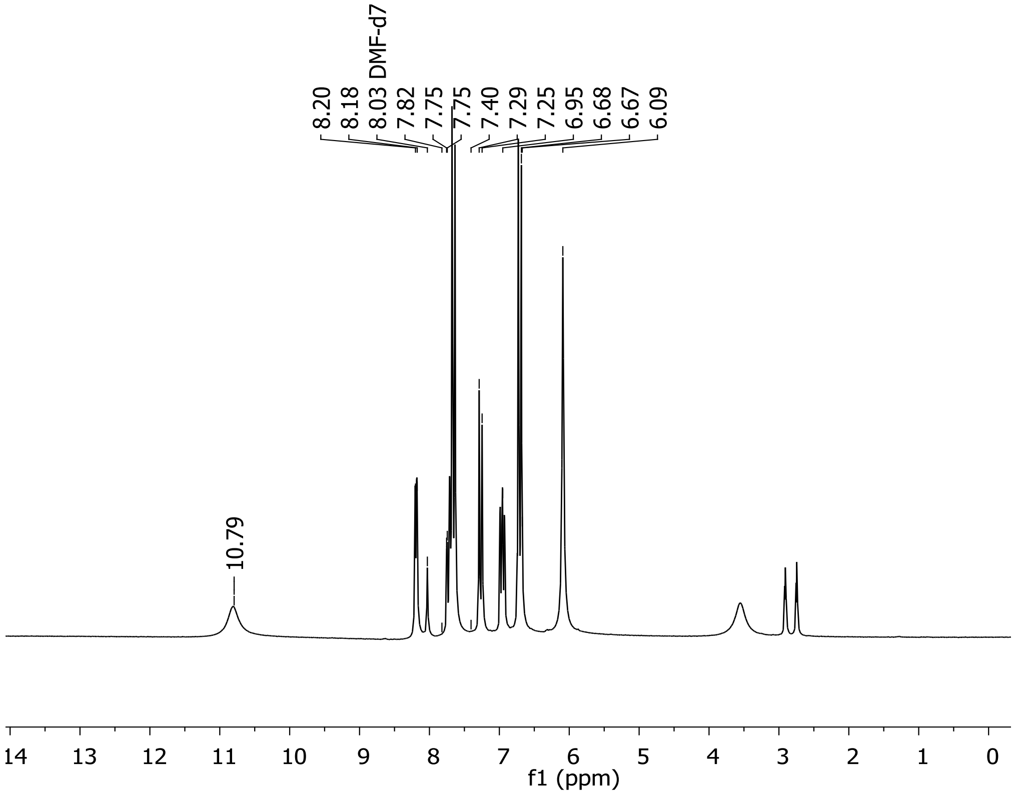

**Figure S3.** *Cont.*

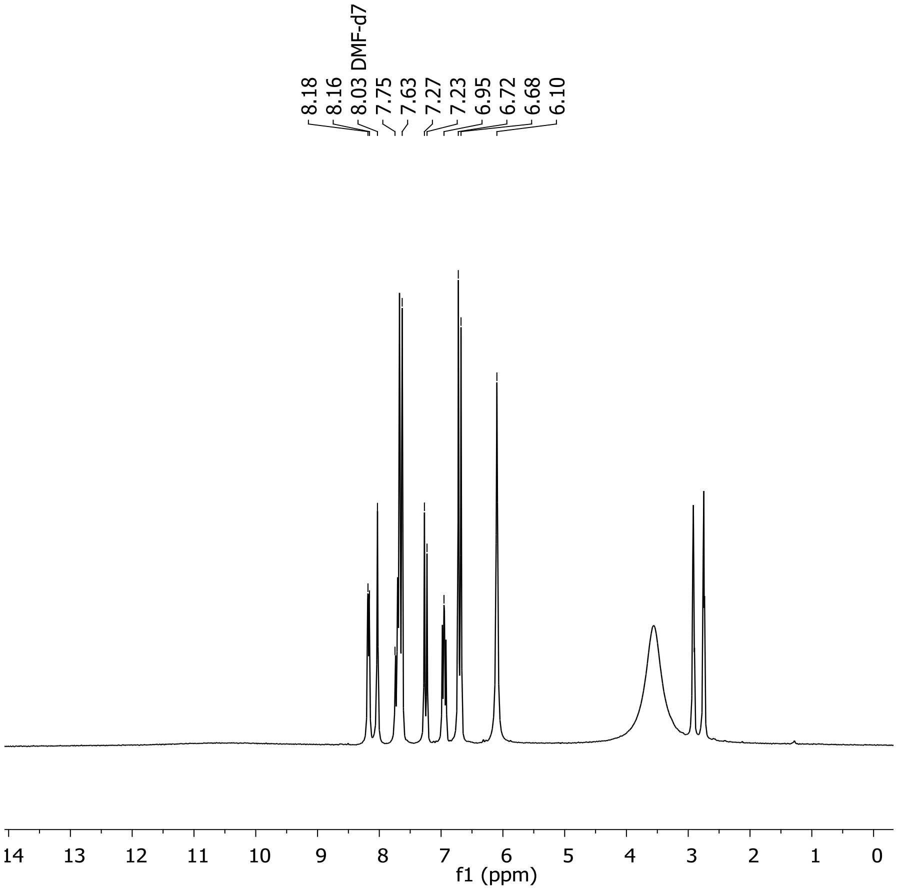

Supplement: Supplementary file 1 [file molecules-18-01464-s002.pdf]
